# 4-Ethyl­amino-3-nitro­benzoic acid

**DOI:** 10.1107/S1600536809014196

**Published:** 2009-04-22

**Authors:** Shivanagere Nagojappa Narendra Babu, Aisyah Saad Abdul Rahim, Shafida Abd Hamid, Ching Kheng Quah, Hoong-Kun Fun

**Affiliations:** a School of Pharmaceutical Sciences, Universiti Sains Malaysia, 11800 USM, Penang, Malaysia; b Kulliyyah of Science, International Islamic University Malaysia (IIUM), Jalan Istana, Bandar Indera Mahkota, 25200 Kuantan, Pahang, Malaysia; c X-ray Crystallography Unit, School of Physics, Universiti Sains Malaysia, 11800 USM, Penang, Malaysia

## Abstract

In the title compound, C_9_H_10_N_2_O_4_, an intra­molecular N—H⋯O hydrogen-bond inter­action generates an *S*(6) ring motif. The nitro group is slightly twisted away from its attached benzene ring [dihedral angle = 15.29 (15)°]. In the crystal structure, mol­ecules are stacked down the *a* axis caused by short O⋯O(−1−*x*, −*y*, 2−*z*) contacts of 2.6481 (16) Å involving the O atoms of the nitro groups. The crystal packing is consolidated by inter­molecular O—H⋯O hydrogen bonds, linking the mol­ecules into centrosymmetric dimers.

## Related literature

For reference bond lengths, see: Allen *et al.* (1987 ▶). For hydrogen-bond motifs, see: Bernstein *et al.* (1995 ▶). For information on the use of derivatives of nitro benzoic acid as precursors for heterocyclic compounds of biological inter­est, see: Ishida *et al.* (2006 ▶). For related structures, see: Mohd. Maidin *et al.* (2008 ▶); Narendra Babu *et al.* (2009 ▶). For the synthesis of ethyl 4-ethylamino-3-nitrobenzoate, see: Li *et al.* (2009 ▶).
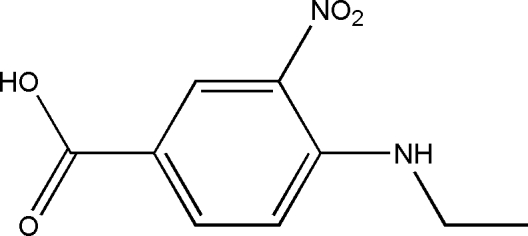


## Experimental

### 

#### Crystal data

C_9_H_10_N_2_O_4_
*M* *_r_* = 210.19
Triclinic, 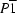

*a* = 3.9354 (4) Å
*b* = 8.4741 (9) Å
*c* = 13.8106 (15) Å
α = 89.256 (5)°
β = 84.730 (4)°
γ = 82.304 (4)°
*V* = 454.49 (8) Å^3^
*Z* = 2
Mo *K*α radiation
μ = 0.12 mm^−1^
*T* = 120 K
0.45 × 0.05 × 0.03 mm

#### Data collection

Bruker SMART APEXII CCD area-detector diffractometer
Absorption correction: multi-scan (**SADABS**; Bruker, 2005 ▶) *T*_min_ = 0.918, *T*_max_ = 0.996
7604 measured reflections
2410 independent reflections
1903 reflections with *I* > 2σ(*I*)
*R*_int_ = 0.023

#### Refinement

*R*[*F* ^2^ > 2σ(*F* ^2^)] = 0.041
*wR*(*F* ^2^) = 0.121
*S* = 1.06
2410 reflections
142 parameters
H atoms treated by a mixture of independent and constrained refinement
Δρ_max_ = 0.44 e Å^−3^
Δρ_min_ = −0.33 e Å^−3^

### 

Data collection: *APEX2* (Bruker, 2005 ▶); cell refinement: *SAINT* (Bruker, 2005 ▶); data reduction: *SAINT*; program(s) used to solve structure: *SHELXTL* (Sheldrick, 2008 ▶); program(s) used to refine structure: *SHELXTL*; molecular graphics: *SHELXTL*; software used to prepare material for publication: *SHELXTL* and *PLATON* (Spek, 2009 ▶).

## Supplementary Material

Crystal structure: contains datablocks global, I. DOI: 10.1107/S1600536809014196/sj2618sup1.cif

Structure factors: contains datablocks I. DOI: 10.1107/S1600536809014196/sj2618Isup2.hkl

Additional supplementary materials:  crystallographic information; 3D view; checkCIF report

## Figures and Tables

**Table 1:** Hydrogen-bond geometry (Å, °)

*D*—H⋯*A*	*D*—H	H⋯*A*	*D*⋯*A*	*D*—H⋯*A*
O4—H1O4⋯O3^i^	0.82	1.80	2.6092 (15)	168
N2—H1N2⋯O1	0.831 (19)	2.052 (18)	2.6634 (15)	130.0 (16)

Symmetry code: (i) 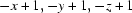.

**Fig. 1.**
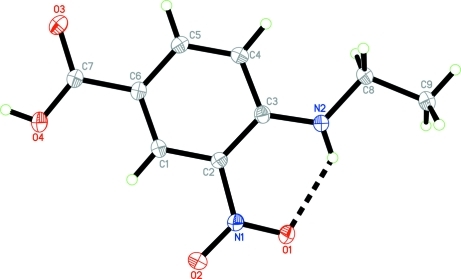
The molecular structure of the title compound, showing 50% probability displacement ellipsoids and the atom numbering scheme. The dashed line indicates an intramolecular hydrogen bond.

**Fig. 2.**
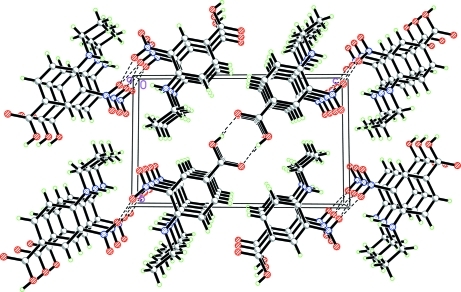
The crystal packing of the title compound,viewed along the a axis. Hydrogen bonds and the short O···O contacts are shown as dashed lines.

## supplementary crystallographic information

### Comment

The derivatives of nitro benzoic acid are convenient precursors for the synthesis of various heterocyclic compounds of biological interest (Ishida *et al.*, 2006). As part of our ongoing studies on new nitro benzoic acid derivatives (Mohd. Maidin *et al.*, 2008; Narendra Babu *et al.*, 2009), we herein present the crystal structure of the title compound.

The molecular structure is stabilized by an intramolecular N2—H1N2···O1 hydrogen bond which generates an S(6) ring motif (Bernstein *et al.*, 1995). The bond lengths (Allen *et al.*, 1987) and angles in the molecule (Fig. 1) are within normal ranges. The nitro groups are slightly twisted away from the attached benzene ring as indicated by the torsion angles O1—N1—C2—C1 and O2—N1—C2—C3 being 165.34 (12)° and 165.04 (12)°, respectively.

In the crystal structure, (Fig. 2), the crystal packing is consolidated by an intermolecular O4—H1O4···O3^i^  hydrogen bond linking the molecules into dimers. There is a short O1···O1 contact (symmetry code: - 1 - *x*, - *y*, 2 - *z*) with distance =  2.6481 (16) which is shorter than the sum of van der Waals radii of the oxygen atoms, stacking the molecules along the *a* axis.

### Experimental

Ethyl 4-ethylamino-3-nitro-benzoate (1.80 g, 0.0075 mol) (Li *et al.*, 2009), and KOH (0.42 g, 0.0075 mol) was refluxed in aqueous ethanol (25 ml) for 3 h. After completion of the reaction, ethanol was distilled off and the reaction mixture was diluted with water (20 ml). The aqueous layer was washed with dichloromethane (10 x 2 ml) and then acidified with concentrated hydrochloric acid to afford a yellow precipitate as the crude product. Recrystallization of the crude product from hot ethyl acetate gave the title compound as yellow crystals suitable for X-ray analysis.

### Refinement

The H-atom attached to N2 was located from the difference Fourier map and refined freely. H atoms of the hydroxy groups were positioned using a rotating group model and constrained with a fixed distance of 0.82 Å. The rest of the hydrogen atoms were positioned geometrically and refined using a riding model with C—H = 0.93–0.97 Å and *U*_iso_(H) = 1.2 or 1.5 *U*_eq_(C). A rotating-group model was also applied for the methyl groups.

### Crystal data

**Table:**

C_9_H_10_N_2_O_4_	*Z* = 2
*M**_r_* = 210.19	*F*(000) = 220
Triclinic, *P*1	*D*_x_ = 1.536 Mg m^−^^3^
Hall symbol: -P 1	Mo *K*α radiation, λ = 0.71073 Å
*a* = 3.9354 (4) Å	Cell parameters from 3018 reflections
*b* = 8.4741 (9) Å	θ = 1.5–29.0°
*c* = 13.8106 (15) Å	µ = 0.12 mm^−^^1^
α = 89.256 (5)°	*T* = 120 K
β = 84.730 (4)°	Needle, yellow
γ = 82.304 (4)°	0.45 × 0.05 × 0.03 mm
*V* = 454.49 (8) Å^3^	

### Data collection

**Table:**

Bruker SMART APEXII CCD area-detector diffractometer	2410 independent reflections
Radiation source: fine-focus sealed tube	1903 reflections with *I* > 2σ(*I*)
graphite	*R*_int_ = 0.023
φ and ω scans	θ_max_ = 29.0°, θ_min_ = 1.5°
Absorption correction: multi-scan (*SADABS*; Bruker, 2005)	*h* = −5→5
*T*_min_ = 0.918, *T*_max_ = 0.996	*k* = −11→11
7604 measured reflections	*l* = −18→18

### Refinement

**Table:**

Refinement on *F*^2^	Primary atom site location: structure-invariant direct methods
Least-squares matrix: full	Secondary atom site location: difference Fourier map
*R*[*F*^2^ > 2σ(*F*^2^)] = 0.041	Hydrogen site location: inferred from neighbouring sites
*wR*(*F*^2^) = 0.121	H atoms treated by a mixture of independent and constrained refinement
*S* = 1.06	*w* = 1/[σ^2^(*F*_o_^2^) + (0.0647*P*)^2^ + 0.1132*P*] where *P* = (*F*_o_^2^ + 2*F*_c_^2^)/3
2410 reflections	(Δ/σ)_max_ < 0.001
142 parameters	Δρ_max_ = 0.44 e Å^−^^3^
0 restraints	Δρ_min_ = −0.33 e Å^−^^3^

### Special details

**Table:**

Experimental. The crystal was placed in the cold stream of an Oxford Cyrosystems Cobra open-flow nitrogen cryostat [Cosier, J. & Glazer, A. M. (1986). J. Appl. Cryst. 19, 105–107] operating at 120.0 (1) K.
Geometry. All e.s.d.'s (except the e.s.d. in the dihedral angle between two l.s. planes) are estimated using the full covariance matrix. The cell e.s.d.'s are taken into account individually in the estimation of e.s.d.'s in distances, angles and torsion angles; correlations between e.s.d.'s in cell parameters are only used when they are defined by crystal symmetry. An approximate (isotropic) treatment of cell e.s.d.'s is used for estimating e.s.d.'s involving l.s. planes.
Refinement. Refinement of *F*^2^ against ALL reflections. The weighted *R*-factor *wR* and goodness of fit *S* are based on *F*^2^, conventional *R*-factors *R* are based on *F*, with *F* set to zero for negative *F*^2^. The threshold expression of *F*^2^ > σ(*F*^2^) is used only for calculating *R*-factors(gt) *etc*. and is not relevant to the choice of reflections for refinement. *R*-factors based on *F*^2^ are statistically about twice as large as those based on *F*, and *R*- factors based on ALL data will be even larger.

### Fractional atomic coordinates and isotropic or equivalent isotropic displacement parameters (Å^2^)

**Table:**

	*x*	*y*	*z*	*U*_iso_*/*U*_eq_	
O1	−0.2816 (3)	0.09151 (12)	0.95790 (7)	0.0267 (3)	
O2	0.0296 (3)	0.28327 (12)	0.93767 (7)	0.0260 (3)	
O3	0.3986 (3)	0.31078 (13)	0.49289 (7)	0.0302 (3)	
O4	0.3740 (3)	0.47173 (12)	0.62170 (8)	0.0272 (3)	
H1O4	0.4493	0.5304	0.5797	0.041*	
N1	−0.0961 (3)	0.17166 (13)	0.90601 (8)	0.0175 (2)	
N2	−0.2305 (3)	−0.12944 (13)	0.82002 (8)	0.0174 (2)	
C1	0.1130 (3)	0.24569 (15)	0.74460 (9)	0.0164 (3)	
H1A	0.1501	0.3413	0.7714	0.020*	
C2	−0.0252 (3)	0.13158 (15)	0.80371 (9)	0.0155 (3)	
C3	−0.0921 (3)	−0.01649 (14)	0.76646 (9)	0.0152 (3)	
C4	0.0068 (3)	−0.04224 (15)	0.66528 (9)	0.0179 (3)	
H4A	−0.0235	−0.1381	0.6374	0.021*	
C5	0.1453 (4)	0.07084 (16)	0.60827 (9)	0.0186 (3)	
H5A	0.2077	0.0496	0.5427	0.022*	
C6	0.1956 (3)	0.21795 (15)	0.64634 (9)	0.0175 (3)	
C7	0.3311 (4)	0.34040 (16)	0.58304 (10)	0.0194 (3)	
C8	−0.2853 (4)	−0.28286 (15)	0.78103 (10)	0.0177 (3)	
H8A	−0.0688	−0.3375	0.7516	0.021*	
H8B	−0.4447	−0.2657	0.7312	0.021*	
C9	−0.4293 (4)	−0.38448 (16)	0.86180 (10)	0.0206 (3)	
H9A	−0.4684	−0.4841	0.8355	0.031*	
H9B	−0.6428	−0.3299	0.8910	0.031*	
H9C	−0.2680	−0.4038	0.9101	0.031*	
H1N2	−0.299 (5)	−0.109 (2)	0.8778 (14)	0.030 (5)*	

### Atomic displacement parameters (Å^2^)

**Table:**

	*U*^11^	*U*^22^	*U*^33^	*U*^12^	*U*^13^	*U*^23^
O1	0.0407 (6)	0.0221 (5)	0.0181 (5)	−0.0142 (4)	0.0083 (4)	−0.0012 (4)
O2	0.0395 (6)	0.0233 (5)	0.0178 (5)	−0.0145 (4)	−0.0011 (4)	−0.0033 (4)
O3	0.0478 (7)	0.0288 (6)	0.0155 (5)	−0.0165 (5)	0.0056 (4)	0.0006 (4)
O4	0.0415 (7)	0.0199 (5)	0.0215 (5)	−0.0142 (4)	0.0048 (4)	0.0009 (4)
N1	0.0222 (6)	0.0146 (5)	0.0155 (5)	−0.0032 (4)	−0.0002 (4)	0.0006 (4)
N2	0.0236 (6)	0.0144 (5)	0.0147 (5)	−0.0057 (4)	0.0004 (4)	0.0001 (4)
C1	0.0179 (6)	0.0142 (6)	0.0176 (6)	−0.0044 (5)	−0.0013 (5)	0.0004 (5)
C2	0.0181 (6)	0.0151 (6)	0.0133 (6)	−0.0025 (5)	−0.0007 (5)	0.0002 (4)
C3	0.0150 (6)	0.0142 (6)	0.0165 (6)	−0.0018 (5)	−0.0023 (5)	0.0011 (5)
C4	0.0215 (7)	0.0165 (6)	0.0162 (6)	−0.0047 (5)	−0.0016 (5)	−0.0022 (5)
C5	0.0215 (7)	0.0204 (6)	0.0143 (6)	−0.0056 (5)	0.0007 (5)	−0.0008 (5)
C6	0.0196 (7)	0.0166 (6)	0.0166 (6)	−0.0050 (5)	0.0002 (5)	0.0014 (5)
C7	0.0232 (7)	0.0192 (6)	0.0166 (6)	−0.0070 (5)	0.0007 (5)	0.0013 (5)
C8	0.0211 (7)	0.0147 (6)	0.0179 (6)	−0.0051 (5)	−0.0016 (5)	−0.0010 (5)
C9	0.0246 (7)	0.0164 (6)	0.0218 (7)	−0.0071 (5)	−0.0016 (5)	0.0007 (5)

### Geometric parameters (Å, °)

**Table:**

O1—N1	1.2375 (14)	C3—C4	1.4273 (18)
O2—N1	1.2273 (14)	C4—C5	1.3700 (18)
O3—C7	1.2700 (17)	C4—H4A	0.9300
O4—C7	1.2784 (16)	C5—C6	1.4043 (18)
O4—H1O4	0.8200	C5—H5A	0.9300
N1—C2	1.4506 (16)	C6—C7	1.4711 (18)
N2—C3	1.3441 (16)	C8—C9	1.5155 (18)
N2—C8	1.4634 (16)	C8—H8A	0.9700
N2—H1N2	0.832 (19)	C8—H8B	0.9700
C1—C6	1.3818 (18)	C9—H9A	0.9600
C1—C2	1.3914 (17)	C9—H9B	0.9600
C1—H1A	0.9300	C9—H9C	0.9600
C2—C3	1.4278 (17)		
			
C7—O4—H1O4	109.5	C4—C5—H5A	119.2
O2—N1—O1	122.64 (11)	C6—C5—H5A	119.2
O2—N1—C2	118.90 (11)	C1—C6—C5	118.53 (12)
O1—N1—C2	118.46 (10)	C1—C6—C7	120.64 (12)
C3—N2—C8	123.74 (11)	C5—C6—C7	120.83 (12)
C3—N2—H1N2	117.9 (13)	O3—C7—O4	123.29 (12)
C8—N2—H1N2	118.3 (13)	O3—C7—C6	118.53 (12)
C6—C1—C2	120.49 (12)	O4—C7—C6	118.18 (12)
C6—C1—H1A	119.8	N2—C8—C9	109.98 (11)
C2—C1—H1A	119.8	N2—C8—H8A	109.7
C1—C2—C3	122.29 (11)	C9—C8—H8A	109.7
C1—C2—N1	115.93 (11)	N2—C8—H8B	109.7
C3—C2—N1	121.78 (11)	C9—C8—H8B	109.7
N2—C3—C4	120.00 (11)	H8A—C8—H8B	108.2
N2—C3—C2	124.66 (12)	C8—C9—H9A	109.5
C4—C3—C2	115.33 (11)	C8—C9—H9B	109.5
C5—C4—C3	121.59 (12)	H9A—C9—H9B	109.5
C5—C4—H4A	119.2	C8—C9—H9C	109.5
C3—C4—H4A	119.2	H9A—C9—H9C	109.5
C4—C5—C6	121.69 (12)	H9B—C9—H9C	109.5
			
C6—C1—C2—C3	−0.9 (2)	N2—C3—C4—C5	179.34 (13)
C6—C1—C2—N1	178.84 (11)	C2—C3—C4—C5	−2.10 (19)
O2—N1—C2—C1	−14.67 (18)	C3—C4—C5—C6	−0.3 (2)
O1—N1—C2—C1	165.34 (12)	C2—C1—C6—C5	−1.6 (2)
O2—N1—C2—C3	165.04 (12)	C2—C1—C6—C7	177.98 (12)
O1—N1—C2—C3	−14.95 (19)	C4—C5—C6—C1	2.2 (2)
C8—N2—C3—C4	0.74 (19)	C4—C5—C6—C7	−177.39 (13)
C8—N2—C3—C2	−177.68 (12)	C1—C6—C7—O3	−178.93 (12)
C1—C2—C3—N2	−178.83 (12)	C5—C6—C7—O3	0.7 (2)
N1—C2—C3—N2	1.5 (2)	C1—C6—C7—O4	0.9 (2)
C1—C2—C3—C4	2.69 (19)	C5—C6—C7—O4	−179.51 (13)
N1—C2—C3—C4	−177.01 (11)	C3—N2—C8—C9	177.29 (12)

### Hydrogen-bond geometry (Å, °)

**Table:**

*D*—H···*A*	*D*—H	H···*A*	*D*···*A*	*D*—H···*A*
O4—H1O4···O3^i^	0.82	1.80	2.6092 (15)	168
N2—H1N2···O1	0.831 (19)	2.052 (18)	2.6634 (15)	130.0 (16)

Symmetry codes: (i) −*x*+1, −*y*+1, −*z*+1.
